# (2*R*,3*R*)-3-*O*-Benzoyl-*N*-benzyl­tartramide^1^

**DOI:** 10.1107/S1600536812022933

**Published:** 2012-05-26

**Authors:** Izabela D. Madura, Janusz Zachara, Urszula Bernaś, Halina Hajmowicz, Ludwik Synoradzki

**Affiliations:** a Warsaw University of Technology, Faculty of Chemistry, Noakowskiego 3, 00-664 Warszawa, Poland

## Abstract

The title compound, C_18_H_17_NO_6_ [systematic name: (2*R*,3*R*)-4-benzyl­amino-2-benzo­yloxy-3-hy­droxy-4-oxobutanoic acid], is the first structurally characterized unsymmetrical monoamide–monoacyl tartaric acid derivative. The mol­ecule shows a staggered conformation around the tartramide C*sp^3^*—C*sp^3^* bond with *trans*-oriented carboxyl and amide groups. The mol­ecular conformation is stabilized by an intra­molecular N—H⋯O hydrogen bond. In the crystal, mol­ecules are linked by O—H⋯O hydrogen bonds between the carboxyl and amide carbonyl groups, forming translational chains along [001]. Further O—H⋯O and N—H⋯O hydrogen bonds as well as weaker C—H⋯O and C—H⋯π inter­molecular inter­actions extend the supra­molecular assembly into a double-layer structure parallel to (100). There are no directional inter­actions between the double layers.

## Related literature

For crystal structures of *R*,*R*-tartaric mono amides, see: Rychlewska *et al.* (1999 ▶); Rychlewska & Warżajtis (2000 ▶, 2001 ▶). For examples of the crystal structures of monoacyl derivatives, see: Madura *et al.* (2010 ▶); Knyazev *et al.* (1988 ▶); Chekhlov *et al.* (1986 ▶); Ishihara *et al.* (1993 ▶). For the synthesis, see: Bell (1987 ▶); Bernaś *et al.* (2010 ▶). 
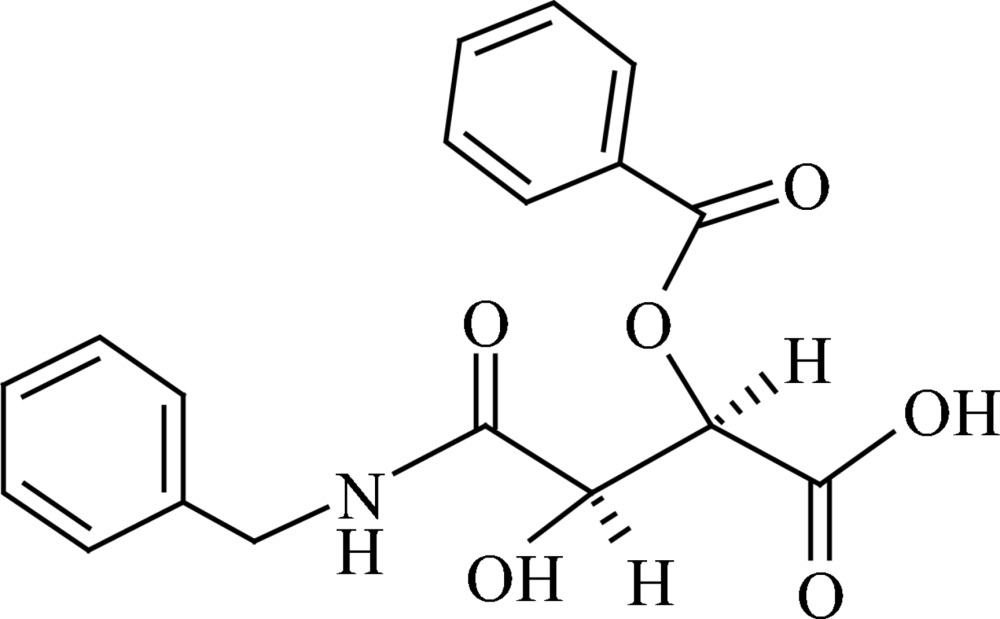


## Experimental

### 

#### Crystal data

C_18_H_17_NO_6_
*M* *_r_* = 343.33
Monoclinic, 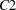

*a* = 35.7118 (6) Å
*b* = 6.17734 (11) Å
*c* = 7.48599 (15) Å
β = 93.0377 (15)°
*V* = 1649.12 (5) Å^3^
*Z* = 4
Cu *K*α radiation
μ = 0.88 mm^−1^
*T* = 100 K
0.54 × 0.22 × 0.18 mm

#### Data collection

Agilent Gemini A Ultra diffractometer
Absorption correction: multi-scan (*CrysAlis PRO*; Oxford Diffraction, 2009 ▶) *T*_min_ = 0.724, *T*_max_ = 1.000
29440 measured reflections
2945 independent reflections
2928 reflections with *I* > 2σ(*I*)
*R*_int_ = 0.038

#### Refinement

*R*[*F* ^2^ > 2σ(*F* ^2^)] = 0.026
*wR*(*F* ^2^) = 0.071
*S* = 1.06
2945 reflections
235 parameters
1 restraint
H atoms treated by a mixture of independent and constrained refinement
Δρ_max_ = 0.19 e Å^−3^
Δρ_min_ = −0.17 e Å^−3^
Absolute structure: Flack (1983 ▶), 1321 Friedel pairs
Flack parameter: −0.01 (13)

### 

Data collection: *CrysAlis PRO* (Oxford Diffraction, 2009 ▶); cell refinement: *CrysAlis PRO*; data reduction: *CrysAlis PRO*; program(s) used to solve structure: *SHELXS97* (Sheldrick, 2008 ▶); program(s) used to refine structure: *SHELXL97* (Sheldrick, 2008 ▶); molecular graphics: *ORTEP-3 for Windows* (Farrugia, 1997 ▶); software used to prepare material for publication: *OLEX2* (Dolomanov *et al.*, 2009 ▶), *PLATON* (Spek, 2009 ▶) and *publCIF* (Westrip, 2010 ▶).

## Supplementary Material

Crystal structure: contains datablock(s) global, I. DOI: 10.1107/S1600536812022933/gk2491sup1.cif

Structure factors: contains datablock(s) I. DOI: 10.1107/S1600536812022933/gk2491Isup2.hkl

Supplementary material file. DOI: 10.1107/S1600536812022933/gk2491Isup3.mol

Supplementary material file. DOI: 10.1107/S1600536812022933/gk2491Isup4.cml

Additional supplementary materials:  crystallographic information; 3D view; checkCIF report

## Figures and Tables

**Table 1:** Hydrogen-bond geometry (Å, °)

*D*—H⋯*A*	*D*—H	H⋯*A*	*D*⋯*A*	*D*—H⋯*A*
O1—H1⋯O5^i^	0.99 (2)	1.55 (2)	2.5317 (13)	171.2 (17)
O4—H4⋯O2^ii^	0.81 (2)	1.96 (2)	2.7501 (14)	165 (2)
N1—H1*A*⋯O6^iii^	0.88 (2)	2.002 (19)	2.7788 (17)	146.4 (18)
N1—H1*A*⋯O4	0.88 (2)	2.12 (2)	2.5894 (14)	112.8 (15)
C12—H12*A*⋯O1^iv^	0.99	2.52	3.4827 (16)	165
C12—H12*B*⋯O1^v^	0.99	2.44	3.3117 (16)	146

Symmetry codes: (i) 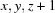; (ii) 
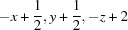
; (iii) 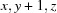; (iv) 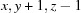; (v) 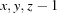.

**Fig. 1.**
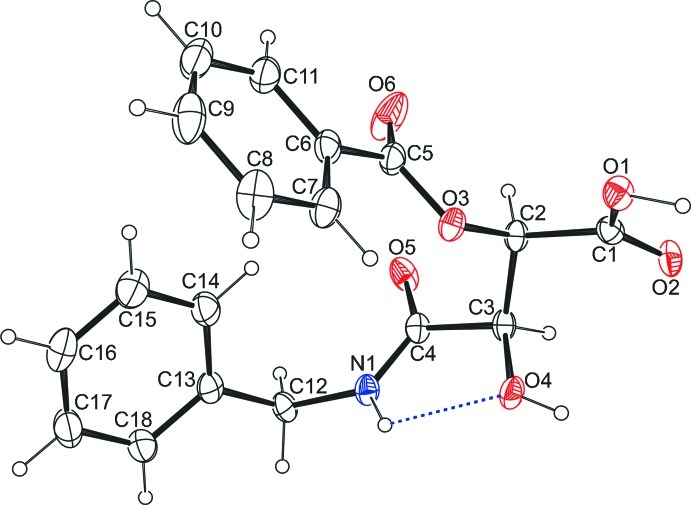
ORTEP plot of the molecular structure of the title compound, with displacement ellipsoids drawn at the 50% probability level. Hydrogen atoms are depicted as spheres with arbitrary radii. Dotted line indicates intramolecular hydrogen bond.

**Fig. 2.**
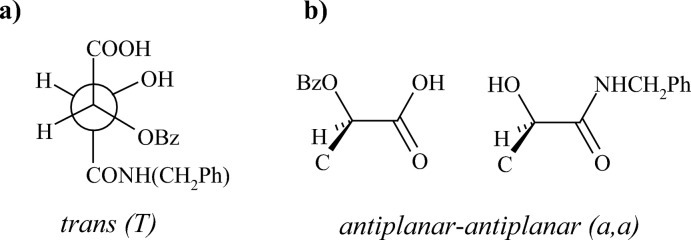
(a) The staggered conformation (T) around C2—C3 bond; (b) Antiplanar (a) conformations around C1—C2 and C4—C3 bonds.

**Fig. 3.**
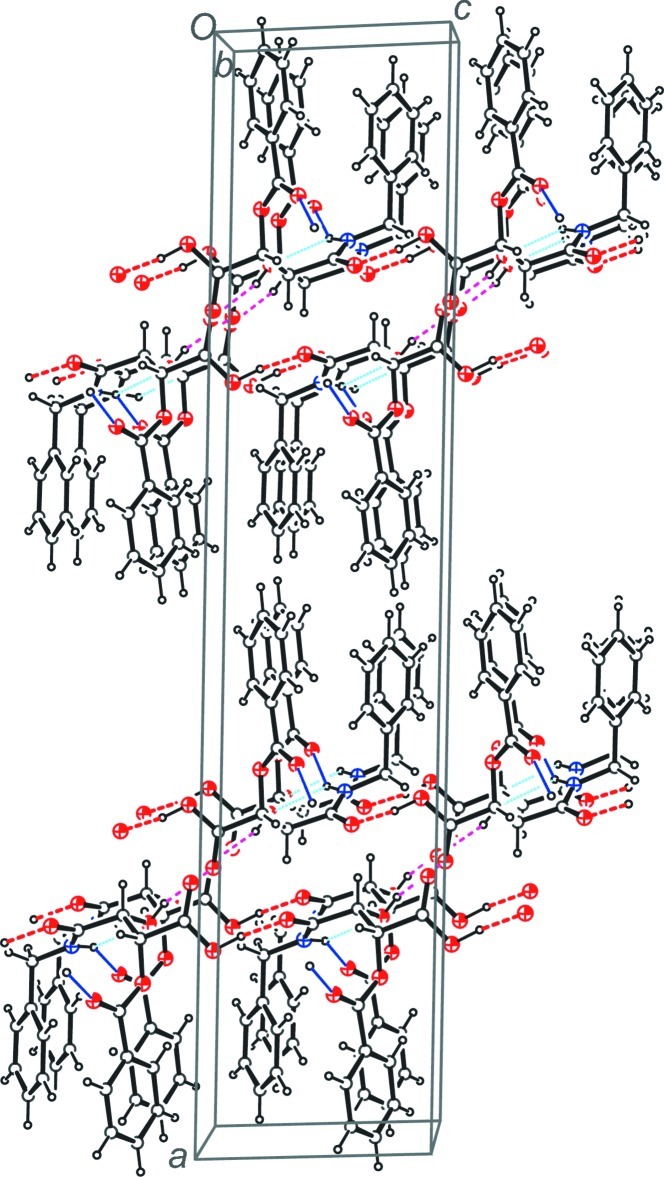
View along the [010] direction showing double-layers of molecules formed by two types of O—H···O (dashed lines) and N—H···O (dotted lines) bonds. Symmetry codes: (i) x, y, z + 1; (ii) -x + 1/2, y + 1/2, -z + 2; (iii) x, y + 1, z.

## supplementary crystallographic information

### Comment

The title molecule crystallizes in non-centrosymmetric *C*2 space group as *R,R* enantiomer. Molecular structure with the atom numbering scheme is presented in Fig. 1. Similarly to the previously characterized tartaric acid mono amides (Rychlewska *et al.*, 1999; Rychlewska & Warżajtis, 2000, 2001), the title molecule adopts the staggered conformation around the C2–C3 bond (Fig. 2.). Thus, the carboxylic group is in *trans* (*T*) orientation with respect to the amide group, whereas the hydroxy and benzoyl substituents adopt the *gauche* counterclockwise orientation. The conformation on C–C* bond in amide fragment enables the formation of an intramolecular N–H···O hydrogen bond between N–H donor and the hydroxyl group. Hence the carbonyl group is on the opposite side of the proximal C*–O bond. Such orientation, called antiplanar (*a*) is also observed in the carboxylic fragment, and the overall conformation of molecules can be given as *T*(*a,a*). It is worth noting that in case of dibenzoyl tartaric mono amides (Rychlewska & Warżajtis, 2001) or those with unsubstituted OH groups (Rychlewska *et al.*, 1999; Rychlewska & Warżajtis, 2000) the conformation of the acid fragment is such that the carbonyl group eclipses the nearest C–O bond (*synplanar* conformation, *s*), while the presence of at least one N–H bond forces the conformation of the amide fragment to be *antiplanar* with the intramolecular N–H···O bond.

The analysis of intermolecular interactions shows that the title molecules related by translation along [001] form infinite head-to-tail chains *via* hydrogen bonds between carboxylic OH donors and amide carbonyl groups. A topologically analogous chain motif was observed in the crystal structures of dibenzoyl mono amides by Rychlewska & Warżajtis (2001). Further, the O–H hydroxyl group acts as a donor to carboxyl C=O group joining the chains related by *2*_1_ screw axis into a double layer (Fig. 3). The layer is enhanced by N—H···O_benzoyl carbonyl_ as well as weaker C—H···O and C—H···π intermolecular interactions. The neighbouring layers are held together by weak van der Waals forces only.

### Experimental

A (1:2 mol/mol) mixture of *O*-benzoyl-*L*-tartaric anhydride (Bernaś *et al.*, 2010) and benzylamine in acetonitrile was stirred at room temperature for 10 min. The mixture was then acidified with 10% HCl and filtered. The resulting white solid product was rinsed with water to give pure title compound with m.p. 465–467 K. Preparation and characterization of regioisomer of the title compound was described by Bell (1987), although its structure was defined incorrectly. [α]^25^_D_ = +40.9°, (c 1, EtOH). IR (EtOH): ν = 693, 708 (C=C, Ph); 1112, 1263 (C—O); 1663 (C=O, CONH); 1710 (C=O, COOH); 1723 (C=O, COBz) cm^-1. 1^H NMR (400 MHz, DMSO-*d6*): δ = 4.43–4.15 (m, 2H), 4.62 (d, 1H), 5.53 (d, 1H), 6.42 (br), 6.81–6.91 (m, 5H), 7.42–7.99 (m, 5H), 8.69 (t, 1H) p.p.m.. ^13^C NMR (400 MHz, DMSO-*d6*): δ = 41.87 (CH2), 71.33 (CH), 74.04 (CH), 126.44, 127.88, 128.76, 128.76, 129.06, 129.52, 133.71, 139.25 (Ph), 165.01, 168.93, 170.28 (C=O) p.p.m.. Anal. Calcd. (%) for C_18_H_17_NO_6_: C 62.97; H 4.99; N 4.08. Found: C 62.92; H 4.99; N 4.11. Crystals suitable for single-crystal X-ray diffraction measurement were obtained from saturated ethyl acetate/methanol (3:1).

### Refinement

The position of the H atoms attached to O and N atoms were freely refined with *U*_iso_(H) = 1.2×*U*_eq_(N) and *U*_iso_(H) =1.5×*U*_eq_(O). Other H atoms were positioned geometrically and refined using a riding model with C—H = 0.95–0.99 Å and with *U*_iso_(H) = 1.2× *U*_eq_(C). The absolute structure was assigned on the basis of anomalous dispersion that confirmed the known chirality of the reagent. The estimated number of measured Friedel pairs amounts to 1321 with the fraction of 0.813.

### Crystal data

**Table:**

C_18_H_17_NO_6_	*D*_x_ = 1.383 Mg m^−^^3^
*M**_r_* = 343.33	Melting point: 193 K
Monoclinic, *C*2	Cu *K*α radiation, λ = 1.5418 Å
*a* = 35.7118 (6) Å	Cell parameters from 23556 reflections
*b* = 6.17734 (11) Å	θ = 5.0–67.0°
*c* = 7.48599 (15) Å	µ = 0.88 mm^−^^1^
β = 93.0377 (15)°	*T* = 100 K
*V* = 1649.12 (5) Å^3^	Prism, colourless
*Z* = 4	0.54 × 0.22 × 0.18 mm
*F*(000) = 720	

### Data collection

**Table:**

Agilent Gemini A Ultra diffractometer	2945 independent reflections
Radiation source: Enhance Ultra (Cu) X-ray Source	2928 reflections with *I* > 2σ(*I*)
Mirror monochromator	*R*_int_ = 0.038
Detector resolution: 10.3347 pixels mm^-1^	θ_max_ = 67.1°, θ_min_ = 5.0°
ω scans	*h* = −42→42
Absorption correction: multi-scan (*CrysAlis PRO*; Oxford Diffraction, 2009)	*k* = −7→7
*T*_min_ = 0.724, *T*_max_ = 1.000	*l* = −8→8
29440 measured reflections	

### Refinement

**Table:**

Refinement on *F*^2^	Secondary atom site location: difference Fourier map
Least-squares matrix: full	Hydrogen site location: inferred from neighbouring sites
*R*[*F*^2^ > 2σ(*F*^2^)] = 0.026	H atoms treated by a mixture of independent and constrained refinement
*wR*(*F*^2^) = 0.071	*w* = 1/[σ^2^(*F*_o_^2^) + (0.0442*P*)^2^ + 0.6346*P*] where *P* = (*F*_o_^2^ + 2*F*_c_^2^)/3
*S* = 1.06	(Δ/σ)_max_ = 0.001
2945 reflections	Δρ_max_ = 0.19 e Å^−^^3^
235 parameters	Δρ_min_ = −0.17 e Å^−^^3^
1 restraint	Absolute structure: Flack (1983), 1321 Friedel pairs
Primary atom site location: structure-invariant direct methods	Flack parameter: −0.01 (13)

### Special details

**Table:**

Geometry. All e.s.d.'s (except the e.s.d. in the dihedral angle between two l.s. planes) are estimated using the full covariance matrix. The cell e.s.d.'s are taken into account individually in the estimation of e.s.d.'s in distances, angles and torsion angles; correlations between e.s.d.'s in cell parameters are only used when they are defined by crystal symmetry. An approximate (isotropic) treatment of cell e.s.d.'s is used for estimating e.s.d.'s involving l.s. planes.
Refinement. Refinement of *F*^2^ against ALL reflections. The weighted *R*-factor *wR* and goodness of fit *S* are based on *F*^2^, conventional *R*-factors *R* are based on *F*, with *F* set to zero for negative *F*^2^. The threshold expression of *F*^2^ > σ(*F*^2^) is used only for calculating *R*-factors(gt) *etc*. and is not relevant to the choice of reflections for refinement. *R*-factors based on *F*^2^ are statistically about twice as large as those based on *F*, and *R*- factors based on ALL data will be even larger.

### Fractional atomic coordinates and isotropic or equivalent isotropic displacement parameters (Å^2^)

**Table:**

	*x*	*y*	*z*	*U*_iso_*/*U*_eq_	
O1	0.31056 (3)	0.28482 (16)	1.07911 (12)	0.0222 (2)	
H1	0.2993 (5)	0.315 (4)	1.194 (3)	0.046*	
O2	0.25203 (3)	0.35877 (17)	0.97437 (12)	0.0229 (2)	
O3	0.33951 (2)	0.36177 (15)	0.77766 (11)	0.0195 (2)	
O4	0.29381 (3)	0.72671 (16)	0.76263 (12)	0.0213 (2)	
H4	0.2772 (6)	0.764 (4)	0.825 (3)	0.046*	
O5	0.28714 (3)	0.38296 (17)	0.38200 (11)	0.0254 (2)	
O6	0.34669 (3)	0.0721 (2)	0.60328 (18)	0.0446 (3)	
N1	0.31211 (3)	0.7200 (2)	0.43257 (14)	0.0196 (2)	
H1A	0.3160 (5)	0.818 (3)	0.517 (3)	0.037*	
C1	0.28496 (3)	0.3271 (2)	0.95296 (16)	0.0185 (3)	
C2	0.29952 (3)	0.3381 (2)	0.76528 (16)	0.0175 (3)	
H2	0.2924	0.2040	0.6969	0.021*	
C3	0.28249 (3)	0.5372 (2)	0.67065 (16)	0.0185 (3)	
H3	0.2545	0.5262	0.6689	0.022*	
C4	0.29469 (3)	0.5431 (2)	0.47812 (16)	0.0176 (3)	
C5	0.36003 (4)	0.2140 (2)	0.69603 (17)	0.0217 (3)	
C6	0.40098 (4)	0.2478 (2)	0.72820 (16)	0.0220 (3)	
C7	0.41579 (4)	0.4261 (3)	0.82131 (18)	0.0256 (3)	
H7	0.3996	0.5298	0.8704	0.031*	
C8	0.45432 (4)	0.4509 (3)	0.8417 (2)	0.0325 (4)	
H8	0.4646	0.5732	0.9037	0.039*	
C9	0.47800 (4)	0.2982 (3)	0.7721 (2)	0.0341 (4)	
H9	0.5044	0.3155	0.7874	0.041*	
C10	0.46321 (4)	0.1209 (3)	0.6808 (2)	0.0327 (3)	
H10	0.4795	0.0163	0.6337	0.039*	
C11	0.42477 (4)	0.0950 (3)	0.6574 (2)	0.0275 (3)	
H11	0.4147	−0.0263	0.5935	0.033*	
C12	0.32616 (4)	0.7696 (2)	0.25671 (16)	0.0202 (3)	
H12A	0.3175	0.9160	0.2196	0.024*	
H12B	0.3154	0.6647	0.1680	0.024*	
C13	0.36848 (4)	0.7612 (2)	0.25630 (16)	0.0206 (3)	
C14	0.38841 (4)	0.5883 (2)	0.33400 (19)	0.0270 (3)	
H14	0.3753	0.4735	0.3876	0.032*	
C15	0.42720 (4)	0.5825 (3)	0.3336 (2)	0.0310 (3)	
H15	0.4406	0.4653	0.3885	0.037*	
C16	0.44647 (4)	0.7487 (3)	0.25267 (19)	0.0308 (3)	
H16	0.4731	0.7458	0.2530	0.037*	
C17	0.42679 (4)	0.9175 (3)	0.1720 (2)	0.0317 (3)	
H17	0.4399	1.0290	0.1141	0.038*	
C18	0.38793 (4)	0.9259 (2)	0.17444 (19)	0.0260 (3)	
H18	0.3746	1.0441	0.1203	0.031*	

### Atomic displacement parameters (Å^2^)

**Table:**

	*U*^11^	*U*^22^	*U*^33^	*U*^12^	*U*^13^	*U*^23^
O1	0.0249 (4)	0.0281 (5)	0.0133 (4)	0.0021 (4)	−0.0010 (3)	0.0001 (4)
O2	0.0203 (4)	0.0337 (5)	0.0152 (4)	0.0001 (4)	0.0047 (3)	0.0039 (4)
O3	0.0167 (4)	0.0254 (5)	0.0165 (4)	0.0000 (4)	0.0009 (3)	−0.0036 (4)
O4	0.0232 (4)	0.0257 (5)	0.0157 (4)	−0.0002 (4)	0.0062 (3)	−0.0049 (4)
O5	0.0358 (5)	0.0284 (5)	0.0123 (4)	−0.0105 (4)	0.0024 (4)	−0.0018 (4)
O6	0.0270 (5)	0.0438 (7)	0.0643 (8)	−0.0105 (5)	0.0154 (5)	−0.0338 (6)
N1	0.0207 (5)	0.0243 (6)	0.0140 (5)	−0.0020 (5)	0.0032 (4)	−0.0020 (5)
C1	0.0228 (6)	0.0175 (6)	0.0152 (6)	−0.0023 (5)	0.0011 (5)	−0.0002 (5)
C2	0.0167 (6)	0.0237 (6)	0.0121 (6)	−0.0015 (5)	0.0010 (4)	−0.0019 (5)
C3	0.0175 (6)	0.0257 (7)	0.0125 (6)	−0.0014 (6)	0.0020 (4)	−0.0010 (5)
C4	0.0151 (5)	0.0248 (6)	0.0128 (6)	0.0000 (5)	−0.0010 (4)	0.0011 (5)
C5	0.0229 (6)	0.0235 (7)	0.0192 (6)	−0.0012 (6)	0.0065 (5)	−0.0028 (6)
C6	0.0223 (6)	0.0284 (8)	0.0156 (6)	0.0019 (6)	0.0046 (5)	0.0034 (5)
C7	0.0234 (7)	0.0343 (8)	0.0192 (6)	0.0007 (6)	0.0030 (5)	−0.0037 (6)
C8	0.0243 (7)	0.0486 (10)	0.0243 (7)	−0.0042 (7)	−0.0010 (6)	−0.0054 (7)
C9	0.0181 (6)	0.0575 (11)	0.0266 (7)	0.0026 (7)	0.0009 (5)	0.0055 (7)
C10	0.0269 (8)	0.0434 (9)	0.0286 (8)	0.0110 (7)	0.0083 (6)	0.0052 (7)
C11	0.0269 (7)	0.0314 (8)	0.0250 (7)	0.0035 (6)	0.0073 (6)	0.0009 (6)
C12	0.0241 (6)	0.0237 (7)	0.0131 (6)	−0.0025 (5)	0.0027 (5)	0.0028 (5)
C13	0.0241 (6)	0.0255 (7)	0.0124 (5)	−0.0019 (5)	0.0029 (5)	−0.0020 (5)
C14	0.0270 (7)	0.0307 (8)	0.0237 (7)	0.0014 (6)	0.0059 (5)	0.0052 (6)
C15	0.0272 (7)	0.0399 (9)	0.0261 (7)	0.0062 (6)	0.0029 (6)	0.0027 (6)
C16	0.0203 (6)	0.0437 (9)	0.0289 (7)	−0.0010 (6)	0.0049 (5)	−0.0088 (7)
C17	0.0289 (8)	0.0345 (8)	0.0329 (8)	−0.0083 (6)	0.0111 (6)	−0.0021 (7)
C18	0.0278 (7)	0.0267 (7)	0.0241 (7)	−0.0017 (6)	0.0055 (6)	0.0019 (6)

### Geometric parameters (Å, º)

**Table:**

O1—C1	1.3053 (15)	C8—H8	0.9500
O1—H1	0.99 (2)	C9—C8	1.387 (2)
O2—C1	1.2111 (16)	C9—H9	0.9500
O3—C2	1.4336 (14)	C10—C9	1.381 (3)
O3—C5	1.3384 (16)	C10—C11	1.384 (2)
O4—C3	1.4067 (17)	C10—H10	0.9500
O4—H4	0.81 (2)	C11—H11	0.9500
O5—C4	1.2442 (17)	C12—H12A	0.9900
O6—C5	1.2010 (18)	C12—H12B	0.9900
N1—C4	1.3115 (18)	C13—C12	1.5126 (17)
N1—C12	1.4659 (16)	C13—C18	1.3922 (19)
N1—H1A	0.88 (2)	C14—C13	1.393 (2)
C2—C1	1.5253 (17)	C14—C15	1.386 (2)
C2—C3	1.5293 (18)	C14—H14	0.9500
C2—H2	1.0000	C15—C16	1.392 (2)
C3—C4	1.5280 (16)	C15—H15	0.9500
C3—H3	1.0000	C16—H16	0.9500
C6—C5	1.4841 (18)	C17—C16	1.379 (2)
C6—C7	1.393 (2)	C17—H17	0.9500
C6—C11	1.393 (2)	C18—H18	0.9500
C7—C8	1.385 (2)	C18—C17	1.390 (2)
C7—H7	0.9500		
			
C1—O1—H1	106.9 (12)	C9—C8—H8	119.8
C5—O3—C2	117.93 (10)	C8—C9—C10	120.05 (13)
C3—O4—H4	108.7 (16)	C8—C9—H9	120.0
C4—N1—C12	126.69 (12)	C10—C9—H9	120.0
C4—N1—H1A	116.4 (13)	C9—C10—C11	120.34 (14)
C12—N1—H1A	116.9 (13)	C9—C10—H10	119.8
O1—C1—O2	125.75 (11)	C11—C10—H10	119.8
O1—C1—C2	114.55 (10)	C6—C11—C10	119.61 (15)
O2—C1—C2	119.70 (11)	C6—C11—H11	120.2
O3—C2—C1	109.39 (9)	C10—C11—H11	120.2
O3—C2—C3	108.56 (10)	N1—C12—C13	112.59 (10)
O3—C2—H2	110.1	N1—C12—H12A	109.1
C1—C2—C3	108.41 (10)	N1—C12—H12B	109.1
C1—C2—H2	110.1	C13—C12—H12A	109.1
C3—C2—H2	110.1	C13—C12—H12B	109.1
O4—C3—C2	110.23 (9)	H12A—C12—H12B	107.8
O4—C3—C4	110.67 (10)	C14—C13—C12	120.92 (12)
O4—C3—H3	108.9	C18—C13—C12	119.84 (12)
C2—C3—H3	108.9	C18—C13—C14	119.23 (12)
C4—C3—C2	109.26 (10)	C13—C14—H14	119.8
C4—C3—H3	108.9	C15—C14—C13	120.48 (13)
O5—C4—N1	127.10 (11)	C15—C14—H14	119.8
O5—C4—C3	117.56 (11)	C14—C15—C16	119.95 (14)
N1—C4—C3	115.34 (11)	C14—C15—H15	120.0
O3—C5—O6	123.49 (12)	C16—C15—H15	120.0
O3—C5—C6	112.86 (11)	C15—C16—H16	120.1
O6—C5—C6	123.63 (12)	C17—C16—C15	119.71 (13)
C5—C6—C7	122.51 (12)	C17—C16—H16	120.1
C5—C6—C11	117.27 (13)	C18—C17—H17	119.7
C7—C6—C11	120.20 (13)	C16—C17—C18	120.58 (14)
C6—C7—C8	119.44 (14)	C16—C17—H17	119.7
C6—C7—H7	120.3	C13—C18—H18	120.0
C8—C7—H7	120.3	C17—C18—C13	120.01 (13)
C7—C8—C9	120.35 (15)	C17—C18—H18	120.0
C7—C8—H8	119.8		
			
C5—O3—C2—C1	123.19 (12)	C11—C6—C5—O3	176.38 (12)
C5—O3—C2—C3	−118.68 (11)	C11—C6—C5—O6	−5.2 (2)
C2—O3—C5—O6	5.3 (2)	C11—C6—C7—C8	0.5 (2)
C2—O3—C5—C6	−176.29 (10)	C5—C6—C7—C8	−178.03 (13)
C12—N1—C4—C3	−178.86 (11)	C5—C6—C11—C10	178.85 (13)
C12—N1—C4—O5	0.9 (2)	C7—C6—C11—C10	0.2 (2)
C4—N1—C12—C13	−108.11 (14)	C6—C7—C8—C9	−0.9 (2)
O3—C2—C1—O1	−16.83 (15)	C10—C9—C8—C7	0.5 (2)
O3—C2—C1—O2	162.45 (12)	C11—C10—C9—C8	0.3 (2)
C3—C2—C1—O1	−135.05 (11)	C9—C10—C11—C6	−0.6 (2)
C3—C2—C1—O2	44.24 (16)	C14—C13—C12—N1	46.90 (17)
O3—C2—C3—O4	−56.88 (12)	C18—C13—C12—N1	−134.32 (13)
O3—C2—C3—C4	64.93 (12)	C12—C13—C18—C17	−179.25 (12)
C1—C2—C3—O4	61.86 (12)	C14—C13—C18—C17	−0.4 (2)
C1—C2—C3—C4	−176.32 (10)	C15—C14—C13—C12	−179.72 (13)
O4—C3—C4—O5	178.58 (11)	C15—C14—C13—C18	1.5 (2)
O4—C3—C4—N1	−1.62 (15)	C13—C14—C15—C16	−1.0 (2)
C2—C3—C4—O5	57.03 (14)	C14—C15—C16—C17	−0.5 (2)
C2—C3—C4—N1	−123.17 (12)	C18—C17—C16—C15	1.6 (2)
C7—C6—C5—O3	−5.05 (18)	C13—C18—C17—C16	−1.1 (2)
C7—C6—C5—O6	173.34 (14)		

### Hydrogen-bond geometry (Å, º)

**Table:**

*D*—H···*A*	*D*—H	H···*A*	*D*···*A*	*D*—H···*A*
O1—H1···O5^i^	0.99 (2)	1.55 (2)	2.5317 (13)	171.2 (17)
O4—H4···O2^ii^	0.81 (2)	1.96 (2)	2.7501 (14)	165 (2)
N1—H1*A*···O6^iii^	0.88 (2)	2.002 (19)	2.7788 (17)	146.4 (18)
N1—H1*A*···O4	0.88 (2)	2.12 (2)	2.5894 (14)	112.8 (15)
C2—H2···O6	1.00	2.25	2.6879 (17)	105
C12—H12*A*···O1^iv^	0.99	2.52	3.4827 (16)	165
C12—H12*B*···O1^v^	0.99	2.44	3.3117 (16)	146

Symmetry codes: (i) *x*, *y*, *z*+1; (ii) −*x*+1/2, *y*+1/2, −*z*+2; (iii) *x*, *y*+1, *z*; (iv) *x*, *y*+1, *z*−1; (v) *x*, *y*, *z*−1.
